# Mechanisms of Comorbidities Associated With the Metabolic Syndrome: Insights from the *JCR:LA-cp* Corpulent Rat Strain

**DOI:** 10.3389/fnut.2016.00044

**Published:** 2016-10-10

**Authors:** Abdoulaye Diane, W. David Pierce, Sandra E. Kelly, Sharon Sokolik, Faye Borthwick, Miriam Jacome-Sosa, Rabban Mangat, Jesus Miguel Pradillo, Stuart McRae Allan, Megan R. Ruth, Catherine J. Field, Rebecca Hutcheson, Petra Rocic, James C. Russell, Donna F. Vine, Spencer D. Proctor

**Affiliations:** ^1^Metabolic and Cardiovascular Diseases Laboratory, Division of Human Nutrition, Alberta Diabetes and Mazakowski Heart Institutes, University of Alberta, Edmonton, AB, Canada; ^2^Department of Sociology, University of Alberta, Edmonton, AB, Canada; ^3^Universidad Complutense de Madrid, Madrid, Spain; ^4^Faculty of Biology, Medicine and Health, University of Manchester, Manchester, UK; ^5^Department of Agricultural Food and Nutritional Science, University of Alberta, Edmonton, AB, Canada; ^6^New York Medical College, New York, NY, USA

**Keywords:** obesity, thrifty genotype, metabolic syndrome, immune function, inflammation, cardiovascular diseases, pcos, JCR rat

## Abstract

Obesity and its metabolic complications have emerged as the epidemic of the new millennia. The use of obese rodent models continues to be a productive component of efforts to understand the concomitant metabolic complications of this disease. In 1978, the *JCR:LA-cp* rat model was developed with an autosomal recessive corpulent (*cp*) trait resulting from a premature stop codon in the extracellular domain of the leptin receptor. Rats that are heterozygous for the *cp* trait are lean-prone, while those that are homozygous (*cp/cp*) spontaneously display the pathophysiology of obesity as well as a metabolic syndrome (MetS)-like phenotype. Over the years, there have been formidable scientific contributions that have originated from this rat model, much of which has been reviewed extensively up to 2008. The premise of these earlier studies focused on characterizing the pathophysiology of MetS-like phenotype that was spontaneously apparent in this model. The purpose of this review is to highlight areas of recent advancement made possible by this model including; emerging appreciation of the “thrifty gene” hypothesis in the context of obesity, the concept of how chronic inflammation may drive obesogenesis, the impact of acute forms of inflammation to the brain and periphery during chronic obesity, the role of dysfunctional insulin metabolism on lipid metabolism and vascular damage, and the mechanistic basis for altered vascular function as well as novel parallels between the human condition and the female *JCR:LA-cp* rat as a model for polycystic ovary disease (PCOS).

## Introduction

### Obesity and the Clinical Problem for Our Generation

Obesity and its metabolic complications have emerged as the epidemic of the new millennia. Fueled by a caloric-dense (and nutrient-poor) food chain that is readily available in most developed countries, the current generation reflects the expression of a human phenotype plagued by the “obesogenic” environment. We have become so efficient at creating foods that target brain reward pathways to stimulate addictiveness and palatability, that we now suffer from the health consequences of not being able to alter our behavior away from this food environment. So too, we are just as guiltily of bestowing this new found talent on the next generation of young adults.

**Figure 1:**
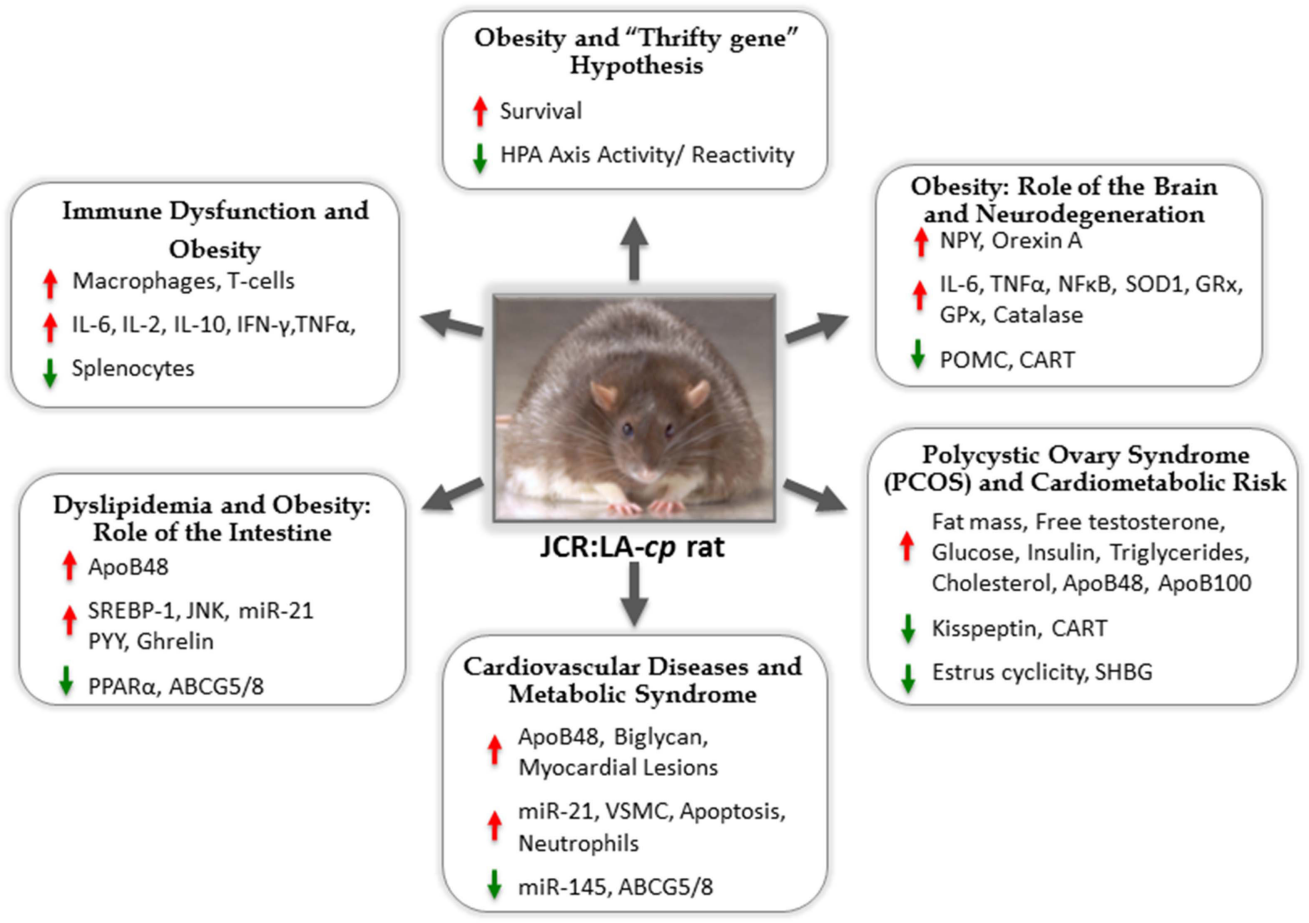
**The *JCR:LA-cp* rat: useful animal model for studies of metabolic syndrome and mechanisms of associated comorbidities**.

For many scientists, this problem has become a life-long commitment to understand how overnutrition and nutrient-poor choices on the backdrop of the existing food chain could result in such a devastating change to our metabolic prognosis in such a short period of time. Animal models have been a prominent tool in this endeavor. One such model, the *JCR:LA-cp* rat (re-derived in Edmonton, AB, Canada in 1978 by Dr. James C. Russell), has received significant attention. For the last three decades, this model has appeared in the literature every year, often in numerous forms spanning facets of nutrition, endocrinology, metabolic syndrome (MetS), obesity, lipid metabolism, vascular myocardial pathophysiology, and pharmacology (Figure 1).

### The *JCR:LA-cp* Rat

In 1978, at the fifth backcross to the LA/N strain [including elements of the corpulent (*cp*) trait], initial breeding stock was sent from National Institutes of Health (NIH) by Dr. Carl T. Hanson to the laboratory of one of the authors (James C. Russell) at the University of Alberta. These rats were the founders of a colony that retained ~3% of the genome derived from the obese spontaneously hypertensive rat (or SHROB). Unlike other NIH colonies, at the time, that were maintained inbred and congenic, the *JCR:LA-cp* strain has been maintained as a closed outbred colony at the University of Alberta to retain the unknown genetic elements leading to early development of cardiovascular disease (CVD).

The formidable contributions that have originated from this rat model have been well documented and reviewed extensively up to 2008 (1–3). The premise of the early studies focused on characterizing the pathophysiology of MetS-like phenotype that was spontaneously apparent in this model. Highlights of the work from this period included useful descriptions of the biochemical profile, careful reports of glucose and insulin metabolism, endothelial function and its impairment, and unique observations of early vascular atherogenesis.

### Contributions of the *JCR:LA-cp* Rat and Recent Advancements

The purpose of this review is to celebrate the major contributions that this rat model has served over many years and to highlight recent advancements. In a unique way, this model has been carefully studied, yet successfully pervasive into the broad areas of research that underpin our understanding of obesity and the MetS.

We wish to highlight areas of advancement made possible by this model including; emerging appreciation of the “thrifty gene” hypothesis in the context of obesity, the concept of how chronic inflammation may drive obesogenesis, the impact of acute forms of inflammation to the brain and periphery during chronic obesity, the role of dysfunctional insulin metabolism on lipid metabolism and vascular damage, the mechanistic basis for altered vascular function as well as novel parallels between the human condition and the female *JCR:LA-cp* rat as a model for polycystic ovary disease (PCOS).

## Obesity: Testing The “Thrifty Gene” Hypothesis of Adaptation to Dietary Energy Intake

The prevalence of obesity is continuing to increase at an alarming rate in both economically advanced Western societies and developing countries in economic–nutrition transition (4). Obesity is linked to adverse short-term and long-term health outcomes, including increased risk of CVD and insulin resistance leading to overt type 2 diabetes (T2D) (5, 6). Genetic differences between individuals explain a major proportion of the within-population variation in body mass index (7). However, genetic susceptibility alone may not result in obesity without other environmental influences (8). A positive energy balance beyond meeting energy requirements results in excess dietary energy intake being preferentially stored as triglycerides (TG) in adipose tissue, resulting in an increase in body weight and fat mass. Obesity can be viewed as a result of physiological dysfunction or perturbation in normal feedback mechanisms relating to feeding behavior and energy balance. The adaptive response or “thrifty gene” theory of obesity explains how genes favoring the efficient use of energy store in periods of feast followed by famine result in obesity in the food-rich or “obesogenic” environment of prosperous societies (9, 10). This “thrifty gene” hypothesis has been the dominant theory of the developmental origins of obesity and related chronic disease. The “thrifty gene” hypothesis provides a potential explanation for the dramatic rate of T2D and obesity prevalence in Pima Indian population of Arizona relative to the genetically similar Pima Indians in Mexico (11). However, there are only limited experimental studies that have tested the adaptive-survival value of an obese-prone genotype. Our ongoing research is based on the *JCR:LA-cp* rodent model that expresses the corpulent (*cp*) autosomal recessive trait (*cp*), a nonsense Tyr763Stop mutation in the Ob-R gene, resulting in a total absence of functional leptin receptors (12). Animals that are homozygous for the *cp* trait (*cp*/*cp*) are obese-prone, hyperphagic, and develop features of the MetS, similar to the metabolic aberrations observed in the clinical setting (13). Animals that are heterozygous for the *cp* trait (+/*cp*) or wild type (+/+) are lean-prone, have normal food intake, and do not develop the MetS. To verify the adaptive response hypothesis that an obese-prone genotype confers a survival advantage when challenged with dietary energy restriction and food-seeking behavior induced activity, juvenile (35–40 days) male *JCR:LA-cp* rats (obese-prone and lean-prone) were exposed to 1.5 h/day of feeding and 22.5 h/day of voluntary wheel running (14). We have shown that this experimental design leads to increased wheel running or food-seeking behavior and self-starvation (14). Initial body weights were similar in both groups of animals; however, the obese-prone animals survived twice as long (8.2 ± 1.1 vs. 3.5 ± 0.2 days) and ran similarly, compared to their lean-prone counterparts (14). A follow-up study showed that prior conditioning with dietary energy restriction for 1 week provided a survival advantage to the obese-prone *JCR:LA-cp* phenotype (15). Dietary energy restriction primed the animals to regulate energy homeostasis pathways following exposure to continued energy restriction and food-seeking-related activity (15). The obese- and lean-prone phenotypes have metabolic differences, which we have shown are associated with alterations in feeding-related neuropeptide gene expression in the arcuate nucleus of the hypothalamus including NPY, Orexin A, CART, and POMC. In addition, hypothalmic pro-inflammatory cytokines and oxidative stress markers IL-6, Tnf-α, and NF-kβ, superoxide dismutase-1 (SOD1), glutathione reductase (GRx), glutathione peroxidase (GPx), and catalase mRNA are elevated in the obese-prone phenotype (16). Interestingly, when the obese- and lean-prone animals are exposed to the same degree of dietary energy restriction, these hypothalamic inflammatory and oxidative stress markers were improved in the obese-prone phenotype, while they are exacerbated in the lean-prone phenotype (16). These findings demonstrate that, in juvenile *JCR:LA-cp* rodents, the metabolic and neural adaptation to energy restriction are dependent on the phenotype, and this can confer a survival advantage (16). Overall, our research findings suggest that obesity is a developmental outcome dependent on the interrelationship of an obese-prone genotype and feeding conditions/“obesogenic” environment. The *JCR:LA-cp* rodent offers a unique opportunity to study the underlying mechanisms of the behavioral and physiological pathways involved in the development of obesity and the possible conference of evolutionary survival traits.

## Characterization of the Altered Immune Function in Obesity Using the *JCR:LA-cp* Rat Model: Modulation with Age and Dietary Fat

Obesity is often recognized as a chronic inflammatory state with altered immune responses, including T cell dysfunction (17–19). The *JCR:LA-cp* rat model of the MetS shares altered immune function, similar to that observed clinically in obese individuals (17–19). The obese-prone rodent model has chronic low-grade systemic inflammation (20–22) and, recently, has been shown to have inflammation in the brain (23, 24). Our group has also demonstrated significant alterations in the acquired immune system of the obese-prone phenotype (19, 25–27). The altered T cell function in the *JCR:LA-cp* rat appears to be dependent on the age of the animal and length of time of exposure to a high-fat diet. When a high-fat diet is introduced at 8 weeks of age and fed for 3 weeks, the obese-prone rats have fewer splenocytes (an indicator of the peripheral lymphopenia) compared to their lean-prone counterparts (27). The obese-prone animals also have a higher proportion of macrophages, T-cells (primarily CD8+ and CD4+) and CD4+CD25+ (T regulatory cells) (27). This inflammatory T cell response would contribute to the overall chronic inflammation observed in these animals. When splenocytes from the *JCR:LA-cp* rats are stimulated *ex vivo* with Concanavalin A (ConA, polyclonal T cell mitogen), they produce the same amount of IL-2 (proliferative cytokine) but have increased levels of the inflammatory cytokine IL-6 (27). In mesenteric lymph nodes (MLN), which are part of the gut-associated lymphoid tissue and visceral adipose tissue, the obese-prone rats have a lower proportion of T-cells (both CD4+ and CD8+ cells) and fewer mature T cells (CD3+CD90+), with a higher proportion of CD4+CD25+ (T regulatory cells) (27). Despite lower T cell numbers, *ex vivo*, MLN from obese-prone rats produce higher amounts of IL-2, IL-10, IFN-γ, and Tnf-α after stimulation with Con A (27), suggestive of a highly reactive pro-inflammatory state in the intestine. When a high-fat diet was fed for 3 weeks to older *JCR:LA-cp* animals (aged 14 weeks), splenocytes had a lower inflammatory response in IL-6 and IL-10 production in the unstimulated condition. In stimulated conditions [with ConA, lipopolysaccharide (LPS), or pokeweed], a lower production of IFN-γ and decreased IL-1β (LPS) and IL-10 (Con A) was observed (25). This suggests, similar to the human condition, that the animals have some degree of immunosuppression and which would make them susceptible to infection. In splenocytes, there was a greater number of CD4+ cells and fewer CD4+CD25+ cells, B cells, and macrophages in obese-prone animals. In MLN, a greater production of IL-4 following Con A stimulation, and IL-1β, IL-10, and IFN-γ after stimulation with LPS was observed (26), again suggestive of intestinal inflammation or increased exposure to antigens (perhaps microbiome) from the intestine. In addition, there was a higher proportion of CD3+CD8+ cells and a lower proportion of CD4+CD25+ in the obese-prone animals (26), consistent with chronic inflammation. When a high-fat diet is introduced at 3 weeks of age and fed for 13 weeks, the obese-prone rats have a lower proportion of CD3+ cells (both CD4+ and CD8+), a higher proportion of CD4+CD25+. In addition, these animals have naive or less mature CD4+ cells and activated B cells in the spleen compared to lean-prone control animals (20), which would leave the animals more prone to infection. With the exception of IL-2 production, which did not differ between obese- and lean-prone animals, the *ex vivo* production of cytokines after stimulation was exacerbated in this group compared to previous studies in older animals or fed for shorter periods of time. After Con A stimulation, splenocytes from obese-prone animals produced lower levels of IL-1β (43%), IL-4 (53%), Tnf-γ (31%), and IFN-γ (31%), and twofold greater IL-6 compared to lean-prone animals (20).

In summary, the obese-prone *JCR:LA-cp* rat displays an immune dysfunction that is similar to that reported in obese individuals (17–19, 28), which suggest lymphopenia and immunosuppression and abnormal inflammatory responses to immune challenges. Differences in immune cell type and function are observed in both the intestinal lymphoid tissue and peripheral immune system suggesting that there is both systemic and intestinal involvement. Interestingly, we have learned that the altered immune profile of the *JCR:LA-cp* rat is also modified by the age and length of feeding period of a high-fat diet. Additionally, we have also demonstrated that immune dysfunction in this model can be improved by dietary supplementation with long-chain polyunsaturated fatty acids (PUFA), including trans-vaccenic acid (20, 27) and fish oil containing docosahexanoic and eicosapentanoic acid (25, 26).

## Inflammation, Stroke, and Infection in Obesity

We and others have contributed to the fact that high levels of circulating biomarkers of inflammation are present in the obese state (29). We know that obese individuals are more likely to develop other chronic inflammatory conditions, including certain forms of cancer, diabetes, cardiovascular, and cerebrovascular disease. Mechanisms by which obesity alters immune and inflammatory responses and how these changes contribute to the development of other diseases are still not clear.

Stroke is a major cause of morbidity and mortality, and the incidence of an ischemic episode has been associated with peripheral and central immune dysfunction (30). Chronic systemic inflammatory conditions, such as infections, atherosclerosis, diabetes, and obesity are associated with increased risk of stroke, suggesting that these conditions and associated inflammation may contribute to the development of stroke (30). In this respect, aged *JCR:LA-cp* rats have been shown to have increased brain inflammation using PET imaging (23). Furthermore, patients with multiple risk factors for stroke, in the absence of any brain pathology, have also been shown to have increased brain inflammation, and this may be associated with increased risk for cerebral ischemia (23). Despite research efforts, there has been a lack of translation of these findings from bench to bed side in stroke patients. One reason for this may be the failure to consider clinical comorbidities in experimental models of stroke. IL-1 is altered peripherally in obesity and is also an important mediator of ischemic brain injury (31). IL-1 receptor antagonist (IL-1Ra) is protective against ischemic brain damage in healthy animals (31). After cerebral ischemia, aged *JCR:LA-cp* rats showed increased blood brain–barrier disruption and brain inflammation compared to their lean-prone counterparts, and this was reduced following systemic administration of IL-1Ra. IL-1Ra treatment was also shown to significantly reduce the infarct volume (measured by MRI) in obese-prone animals, further supporting IL-1Ra as a lead candidate for the treatment of ischemic stroke (24).

Bacterial infections have been proposed to contribute to stroke development and may worsen ischemic event outcomes (32). In this setting, a sustained pulmonary infection (*Streptococcus pneumoniae* isolate) induced in *JCR:LA-cp* was used to investigate the effect of infection on vascular and inflammatory responses prior to and after cerebral ischemia (33). The results showed that the pneumonia infection augmented atherosclerosis and exacerbated ischemic brain injury *via* IL-1 and platelet-mediated systemic inflammation pathways (33). Targeting these mechanisms could be therapeutically useful to prevent infection-induced thrombo-inflammatory responses that may predispose individuals to ischemic vascular events and adversely affect stroke outcomes.

## Development of Dyslipidemia During Obesity and Hyperinsulinemia: Overproduction of Lipids by the Intestine and Bioactive Trans-Fatty Acids

### Intestinal Contribution to Hypercholesterolemia and Diabetic Dyslipidemia

One of the most significant advances that have occurred over the last 5 years with respect to intestinal lipid metabolism is the understanding of how the gut contributes to whole body cholesterol homeostasis (34). In particular, we now appreciate how these intestinal-based mechanisms become dysfunctional under conditions of obesity and insulin resistance. Using the *JCR:LA-cp* rat model, we have shown that circulating hyperinsulinemia (as is the case during conditions of MetS or prediabetes) can lead to increased absorption of both lipid and sterol from intestine that can, in turn, exacerbate dyslipidemia and CVD risk. Specifically, we have demonstrated that insulin resistance results in a chronic overproduction and excessive secretion of intestinal-derived apoB48-containing chylomicron particles. This finding is consistent with studies from the *JCR:LA-cp* rat (35), the hamster (36), and, more recently, humans (37).

Mechanisms responsible for the overproduction of intestinal-derived lipoproteins have been reviewed elsewhere (38) but can include stimulation by excess free fatty acids, manipulation of both GLP-1 and GLP-2, increased circulating Tnf-alpha, increased stability of apoB48, and the downregulation of enterocytic insulin receptor (IR) substrate-1. Work from our own group utilizing the *JCR:LA-cp* rat has also confirmed that this is likely multifactorial resulting from; suppression of enterocytic Pparα (39), increased SREBP-1 (40), decreased ABCG5/G8 (39), and increased phosphorylation of JNK (41). New emerging data also suggest that increased enterocytic Tnfα may also contribute to increased apoB *per se* (42). In addition, Parnell and Reimer have elegantly shown that there is an increase in satiety hormone (proglucagon, PYY, and ghrelin) mRNA expression in the *JCR:LA-cp* rat gut that may potentially contribute to intestinal lipid overproduction (43).

Importantly, these observations provide valuable mechanistic explanation for why those with the MetS and diabetes typically have a unique lipoprotein phenotype. Left untreated, those with obesity and early insulin resistance develop atherogenic dyslipidemia that renders a significantly increased CVD risk profile. Other efforts from our laboratory have used the *JCR:LA-cp* rat to better understand potential nutritional means to curb these metabolic impairments.

### Impact of Dietary Long-Chain Fatty Acids, PUFA, and Ruminant Trans-Fatty Acids to Lipoprotein Metabolism in the *JCR:LA-cp* Rat

Pharmaceutical compounds that activate peroxisome proliferator-activated receptor (PPAR)-α and PPAR-γ (e.g., fibrates and thiazolidinedione, respectively) have been successful as potent lipid-lowering and insulin-sensitizing therapies for CVD and diabetes-related dyslipidemia (44, 45). Activation of PPAR-α can effectively reduce plasma hypertriglyceridemia possibly *via* modulating fatty acid oxidation and energy homeostasis pathways. Upregulation of PPAR-γ activity has also been shown to normalize insulin sensitivity, improve lipid metabolism, and the clearance of lipoproteins, as well as restore vascular contractility and endothelial function (46). We became interested in the fact that a number of natural PPAR-α ligands exist among which include long chain and PUFA (e.g., oleic acid, arachadonic acid, eicosapentaenoic acid, and docosahexaenoic acid) (47, 48). More specifically, conjugated linoleic acid (CLA) is another naturally occurring agonist of the PPAR-α pathway, which has been proposed to be a primary mechanism *via* which CLA elicits pleiotropic effects (49, 50). It has now been recognized that aside from being converted to *c*9,*t*11-CLA *in vivo*, its precursor vaccenic acid (VA) may also have independent bioactivity in regulating lipid metabolism. However, few studies have attempted to explore the metabolic pathways that are potentially modulated by VA. Using the obese *JCR:LA-cp* rat, we have offered a number of contributions to better understand the potential role of VA in activating and thereby regulating lipid metabolism (51, 52). Most recently, our group has obtained data indicating that VA is a potent PPAR-α and PPAR-γ agonist and strongly binds to the ligand-binding domain of both receptors. In accordance, we found a substantial elevation in energy expenditure during both light and dark cycle in VA-supplemented obese *JCR:LA-cp* rats (40, 53). The observed change in energy metabolism may partially be accredited to enhanced citrate synthase activity (an indicator of fatty acid oxidation) in liver and adipose tissue of VA/CLA-fed obese rats. Our evidence implies an active involvement of these nuclear receptors in regulating lipid metabolism, which is shared by other bioactive fatty acids such as DHA and *c*9,*t*11-CLA. These findings have been important in understanding the role of ruminant derived trans-fatty acids as a class, and how they differ from pro-inflammatory industrial produced trans-fatty acids through the partial hydrogenation of vegetable oils (54).

In addition to PUFA and ruminant trans-fatty acids, prebiotic fibres have also been proposed to promote weight loss and lower plasma lipids; yet, the mechanisms are not fully understood. By using the *JCR:LA-cp* rat, Parnell and Reimer have demonstrated that a 10% dietary blend of prebiotic fibres (inulin and oligofructose) can lower total cholesterol concentrations. The dietary blend is thought stimulate cholesterol excretion in the form of bile as well as reduce hepatic steatosis through a FAS independent mechanism (55).

## Arteriogenesis is Mediated by microRNA in the Cardiovasculature and is Associated with Intestinal Lymphatic Lipoproteins in the Metabolic Syndrome

Coronary collateral growth (arteriogenesis) is an important adaptive process in transient, repetitive coronary artery occlusion, and myocardial ischemia, which occurs in stable *angina pectoris*. Well-developed collateral networks are associated with lower incidence and severity of myocardial infarction (56). In contrast to angiogenesis, which is characterized by new vessel (capillary tube) formation, arteriogenesis is defined by remodeling and enlargement of small arterioles (with very low blood flow) to connect with larger conducting arteries. This process critically depends on both normal endothelial (57, 58) and vascular smooth muscle cell (VSMC) function (59). Arteriogenesis has shown to be severely impaired in animal models exhibiting endothelial dysfunction, including the Zucker obese pre-diabetic (ZOF) rat (60) and the MetS obese-prone *JCR:LA-cp* rat (59, 61, 62). Human patients with impaired endothelial function and the MetS also exhibit impaired arteriogenesis (63–65).

We have recently shown that microRNA (miR)-145 and miR-21 are important regulators of arteriogenesis. miR-145 levels are reduced in the *JCR:LA-cp* obese-prone rat compared to the lean-prone metabolically normal animal, and when miR-145 levels are upregulated in the obese-prone animal, the result is complete restoration of coronary collateral growth (59). This effect was mediated by conversion of the aberrant synthetic VSMC phenotype to the normal contractile VSMC phenotype in the obese-prone model (59). miR-21 levels were markedly increased in the heart of obese-prone animals, shown to be positively correlated with VSMC proliferation (66) and decreased apoptosis of neutrophils (66). Conversely, downregulation of miR-21 to levels found in lean-prone animals resulted in significant collateral growth recovery in obese-prone animals, associated with a decrease in VSMC proliferation (66); concomitant with a restoration of apoptosis of neutrophils (67). miR-21 is well accepted as a major pro-survival and pro-proliferative miR. Moreover, elevated miR-21 levels were shown to be positively correlated with: increased expression of pro-proliferative markers (G1/S and G2/M cyclins and cyclin-dependent kinases); low expression of “tumor suppressors” (p21, p27, and pRb); high expression of anti-apoptotic Bcl-2/Bcl-2 dimers; low expression of pro-apoptotic Bcl-2/Bax dimers (caspases 9 and 3); and decreased cytochrome *c* release from the mitochondria. Collectively, this study established that miR-21 can regulate neutrophil apoptosis and is a required component for successful collateral enlargement.

### A Role for the Lymphatic Expression of microRNA in Lipid Metabolism?

Interestingly, miR-21 expression was also significantly increased in jejunal enterocytes isolated from obese-prone *JCR:LA-cp* rat. The concentration of miR-21 in these animals was also increased in intestinal lymph and in the high-density lipoprotein (HDL) fraction isolated from the lymph (66). miR-21 levels in enterocytes were examined because, in the obese-prone animal, the intestine has overproduction of lipids associated with increased secretion of chylomicrons (CM), and HDL is also found in the lymphatics isolated from the intestine (68). This intestinal secretion of lipids contributes significantly to the lipid–lipoprotein content of the lymphatics (68). Our results further indicated that 95% of miRs in intestinal lymph was associated with the HDL fraction, rather than the CM fraction (68). HDL has also been identified as a major transporter of miRs in the circulation (69, 70). More recently, the lymphatic system was revealed to be critical for the metabolic turnover of HDL and the reverse cholesterol transport system (71). These results suggest that altered enterocyte lipid and lipoprotein metabolism and/or HDL-dependent lymphatic cholesterol transport may be inter-related to the elevated cardiac miR-21 levels observed in the obese-prone *JCR:LA-cp* model.

## Early Intimal Atherogenesis, Arterial Lipid Retention, and Novel Therapeutical Targets

### Impact of Remnant Dyslipidemia to Atherosclerotic Vascular Disease during Obesity and Insulin Resistance

One of the major complications of obesity and T2D is the dramatic increased risk for CVD [specifically, atherosclerotic vascular disease (ASVD)], the reasons for which are multifactorial. We have been very interested in understanding the role that chronic metabolic disease has in exacerbating dyslipidemia and how this mechanistically translates into greater lipid deposition within the arterial wall. Atherogenic cholesterol-dense lipoproteins (for example, low-density lipoprotein, LDL-C) are thought to permeate both intact and/or damaged arterial endothelium, become entrapped within the sub-endothelial space, and accumulate, resulting in inflammation and atheroma (72, 73). Although the literature documents a significant epidemiological and/or genetic (i.e., GWAS) association between raised circulating fasting LDL-C and ASVD risk, a large proportion of subjects diagnosed with CVD are either normolipidemic (normal levels of LDL) or have substantial residual risk (74–76). These data suggest that clinically, atheromata-associated cholesterol is derived from alternate sources, including non-fasting remnant lipid fractions as originally proposed by Zilversmit (77). Indeed, the International Atherosclerosis Society has recognized non-fasting measurements of remnant cholesterol as a major target in their “Global Recommendations for the Management of Dyslipidemia” (78).

Interestingly, a series of recent publications authored by the Copenhagen Heart Study group have provided evidence for a major shift in the paradigm of atherogenesis (and potentially its therapy), suggesting that remnant (non-fasting) cholesterol is a major causative factor for ischemic heart disease (IHD) (79, 80).

Using the *JCR:LA-cp* rat, we have also collected a substantial array of preclinical data showing that remnant lipoproteins carry substantially more cholesterol (due to their size and enrichment) compared to other atherogenic fractions. Using well-established arterial perfusion methodology, we have conducted comparative dual labeling lipoprotein experiments. *Cy5*-labeled remnants were isolated, purified, and perfused simultaneously with *Cy3*-labeled LDL (designed to expose equivalent LDL-derived apoB100 and remnant-derived apoB48). We observed a significantly greater number of LDL particles delivered to the vessel (4.5 ± 1 × 10^−9^ µg/µm^2^ tissue) as compared to remnants (0.48 ± 0.15 × 10^−9^ µg/µm^2^ tissue) (81). However, after extensive washout (a further 60 min) with lipoprotein-free buffer (i.e., representing residual *retention*), we observed significantly fewer (55% decrease) LDL particles remaining in the tissue (2.9 ± 0.42 × 10^−9^ µg/µm^2^ “retention” vs. 4.5 ± 1 × 10^−9^ µg/µm^2^ “delivery,” *p* < 0.05). Additional studies have gone on to show that there is an increased deposition of remnant lipoproteins in arteries from *JCR:LA-cp* rats as demonstrated by arterial perfusion of equivalent numbers of particles. Mechanistically, we have proposed that this phenomenon may be due to (a) dyslipoproteinemia and/or (b) perturbations in the vessel wall. Crossover perfusion experiments have revealed that increased retention of the *number of remnant lipoproteins* during insulin resistance was due to differences in arterial vasculature and independent of other factors effecting particle dysfunction *per se*.

### Impact of Obesity and Insulin Resistance on the Extracellular Matrix and Arterial Remodeling

Our group has also shown that intestinal-derived remnant lipoproteins can colocalize with arterial biglycan in an insulin-deficient model of type I diabetes *ex vivo* (82). Subsequently, we have demonstrated, in the *JCR:LA-cp* rat, that in the prediabetic milieu, aortic biglycan protein core content increases significantly with age and correlates linearly with increasing hyperinsulinemia. We know that the expression of biglycan protein core has been shown to be increased with fatty acids (83, 84), angiotensin II (85), and transforming growth factor-β (TGF-β) (86, 87). Consistent with this, obese *JCR:LA-cp* rats have been shown to have elevated concentrations of TGF-β (88) and non-esterified free fatty acids (51).

### Arterial Retention of Remnant Lipoproteins and Associated Cholesterol Deposition in Response to Ezetimibe and Simvastatin

Ezetimibe (EZ) is a pharmaceutical compound that selectively reduces intestinal cholesterol absorption by inhibiting the Niemann-pick C1-like 1 (NPC1L1) transporter (89) while Simvastatin (SV) is a HMG-CoA reductase inhibitor. Using the arterial perfusion approach in the *JCR:LA-cp* rat, we have shown that EZ treatment can ameliorate the deposition of arterial remnants and associated cholesterol *ex vivo* (90). It is also intriguing, that the addition of SV to EZ appeared to have an additional benefit reducing arterial cholesterol deposition, suggesting a synergism of independent modes of action.

### Combination of Ezetimibe with Simvastatin Improves Fasting and Postprandial Lipids

A study by Bozzetto et al. reported that the combination of EZ with SV in T2D subjects can beneficially impact both fasting and postprandial triglyceride-rich lipoproteins (91). They found that the addition of EZ to SV reduced the number of circulating CM particles (apoB48) in the postprandial state, while also lowering both fasting and postprandial chylomicron cholesterol (88). Postprandial data from studies in *JCR:LA-cp* rats are consistent with benefits of either EZ + SV therapy on both remnant particle metabolism and corresponding cholesterol (81).

Ischemic myocardial lesions constitute a critical end point in CVD. Previous studies using the *JCR:LA-cp* rat strain have demonstrated a correlation between the frequency of myocardial lesions with hyperinsulinemia (92). Hearts isolated from *JCR:LA-cp* rats treated with either EZ (−84%) or EZ + SV (−84%) have displayed a significant reduction in the frequency of early stage 2 myocardial lesions or very recent ischemic lesions undergoing scavenging and repair, characteristic for this strain at this age.

## Establishing *JCR:LA-cp* Rodent Rat as a Model of Spontaneous Left Ventricular Heart Dysfunction

The *JCR:LA-cp* rat has previously been identified as a model that also includes pathological complications of endothelial dysfunction and myocardial ischemia, in addition to other dysfunctional complications consistent with the MetS (41, 92, 93). We have recently demonstrated that *JCR:LA-cp* rats exhibit significant cardiac dysfunction and present as a useful animal model of spontaneous LV dysfunction (94). *JCR:LA-cp* rats were subjected to Doppler echocardiography analysis (*Vevo 770 Micro-Imaging system*). 2-D parasternal long- and short-axis images of the left ventricle (LV) were obtained using a 25-MHz linear-array transducer and doppler probe in anatomical M-mode at the level of papillary muscles at a sweep speed of 150 mm/s. We observed that *JCR:LA-cp* rats exhibited distinct signs of cardiac dysfunction, and that hearts exhibited a marked increase (~40%) in LV mass vs. their lean counterparts. Echocardiographical analysis also revealed that hearts from *JCR:LA-cp* rats had an increased early (MV-E) filling velocity (~40%) and reduced late (MV-A) filling (~15%) velocity compared to age-matched lean rats. We further revealed that *JCR:LA-cp* rats had a restrictive filling pattern, shown by a significantly shortened isovolume relaxation time (IVRT) (~30% decrease). Finally, we found that *JCR:LA-cp* rats exhibited progressive worsening of diastolic filling properties, with a 1.6-fold increase in the ratio of early to late filling velocity (E/A).

## The Female *JCR:LA-cp* Rat as a Model of Polycystic Ovary Syndrome and Cardiometabolic Risk

### The *JCR:LA-cp* Rat as a Spontaneous Model of PCOS

Polycystic ovary syndrome (PCOS) has become an increasing public health concern given its association with menstrual dysfunction, infertility, MetS, T2D, and CVD risk (95, 96). The syndrome afflicts 5–18% of women in their reproductive years, in adolescents to premenopausal women. PCOS is diagnosed by the presence of clinical or biochemical hyperandrogenemia, menstrual irregularity, and/or polycystic ovaries (97). The incidence of PCOS is twofold to threefold greater in overweight-obese adolescents and women and is coassociated with features of the MetS including obesity, impaired insulin sensitivity, and dyslipidemia predisposing women to increased risk of prematurely developing T2D and CVD (96, 98, 99). Animal models of PCOS have been used to further understand the role of androgens, in particular testosterone, on the development of the metabolic aberrations in this condition, and the features of these models have been previously reviewed (100, 101). These models have primarily used testosterone to induce PCOS; however, the *JCR:LA-cp* rodent model is the only model to spontaneously develop PCOS in conditions of the MetS (102). The significance of the *JCR:LA-cp* rat in this context is the similarity to the human condition in which the development of the PCOS phenotype is preceded by increased adiposity and insulin resistance. Interestingly, we have known, for some time, that female homozygous *JCR:LA-cp* rats are infertile but have only recently begun to appreciate the metabolic impactions. In humans, females that carry a predisposition for insulin resistance due to family history often see a clinical presentation that antagonizes endocrine–reproductive dysfunction, a feature that is also observed in female *JCR:LA-cp* rats (96, 103, 104).

### Cardiometabolic Risk, Dyslipidemia, and the Hypothalamic–Pituitary–Gonadal Axis

The pathogenesis of PCOS is linked to altered hypothalamic–pituitary–gonadal axis function and perturbed insulin and testosterone metabolism (105, 106). One of the major areas of research in the PCOS-prone *JCR:LA-cp* rodent model is understanding the distinct mechanisms of androgens and insulin in the cardiometabolic manifestations, particularly dyslipidemia and CVD risk (102, 107, 108). Dyslipidemia occurs in greater than 70% of PCOS patients and is positively correlated with increasing quartile of plasma hyperandrogenemia (97, 99). We have characterized the dyslipidemic profile of the PCOS-prone *JCR:LA-cp* rodent model, which has markedly elevated fasting and non-fasting plasma TG, total cholesterol (TC), apoB48, and apoB100 (markers of intestinal CM and hepatic very low-density lipoprotein and low-density lipoproteins, respectively) compared to control animals (35, 104). PCOS-prone animals have twofold the intestinal triglyceride, cholesterol, and apoB48 secretion in the fasted state compared to their lean-prone control counterparts, and this is associated with increased mRNA expression of SREBP-2, LDLR, and apoB (102, 107). When given dietary lipid, this elevated CM lipoprotein particle (apoB48) and lipid (cholesterol and TG) secretion is further exacerbated (107). We have further shown, in this model, that plasma testosterone and insulin are positively correlated with fasting and non-fasting plasma TG and apoB48, consistent with the role of these lipogenic mediators in the development of dyslipidemia in PCOS, insulin resistance, and obesity (106, 107, 109).

Intervention with flutamide, an androgen receptor (AR) inhibitor, has confirmed that testosterone action *via* the AR mediates apoB-hyperlipoproteinemia and hypertriglyceridemia, and this appears to be independent of effects on insulin (110). Fasting plasma apoB100, apoB48, and TG concentrations were lowered by 25–50% in animals treated with flutamide. Flutamide–metformin combination treatment similarly lowered these parameters; however, metformin treatment alone had no effect on fasting plasma lipids, indicating a predominant effect of the AR inhibitor to mediate lowering of plasma lipids. Additionally, the intestinal secretion of TG, cholesterol, and the cholesterol/apoB48 and TG/apoB48 (a marker of lipid per CM particle secreted from the intestine) were markedly reduced following flutamide treatment. Hepatic and intestinal lipogenic gene expression showed that flutamide may lower hepatic SREBP-1, LDLR, and HMGCR in PCOS-prone animals (110). Interestingly, PCOS-prone animals have reduced IR, MAPK1, AKT2, and PTPN1 mRNA expression in the intestine, but not the liver. However, flutamide and metformin treatment appeared to favor hepatic upregulation of the IR mRNA, as well as MAPK1 and protein kinase B (AKT2); however, in the intestine, MAPK1 was downregulated, and no effect on AKT2 mRNA expression was observed in PCOS-prone animals. Overall, these findings indicate that lipogenic and insulin signaling gene expression is altered in PCOS-prone animals compared to lean-prone controls. Effects of AR inhibition and insulin-sensitizing treatments appear to modify these pathways in association with improvements in plasma and intestinal secretion of lipids (110).

### Energy Restriction and Exercise Intervention in the Female *JCR:LA-cp* Rat

In the PCOS-prone model, we have shown that energy restriction and voluntary exercise intervention (4 h/day) at an early life stage can significantly attenuate reproductive and cardiometabolic aberrations (111). The combination of diet and exercise was shown to lower total body weight gain and body fat mass by 30% in PCOS-prone animals. Consistent with our ongoing studies on dietary energy restriction and food-seeking-induced exercise (112, 113), we have also found that energy restriction independently induces food-seeking behavior related activity in the PCOS-prone animal, favoring an increase in energy expenditure. In terms of cardiometabolic risk, the combination of energy restriction and exercise decreased fasting plasma TG and apoB48 in PCOS-prone animals. In addition, the combination of exercise and dietary energy restriction increased serum hormone-binding globulin and free androgen index, and normalized mRNA expression of hypothalamic CART and Kisspeptin. Collectively, these findings were associated with improvements in follicular morphology and estrus cyclicity. In similar exercise conditions, we have shown that the addition of metformin–flutamide treatment lowers total body weight and body fat-pad weight and tends to lower fasting plasma lipids. Interestingly, a combination of both metformin and flutamide treatment, in addition to exercise further reduces free Testosterone and improves estrus cyclicity compared to exercise alone (113).

The findings of this work have revealed voluntary exercise has modest effects on cardiometabolic risk factors, and the inclusion of medications that specifically target insulin resistance and dyslipidemia are required to modulate these risk factors in this obese PCOS-prone model. These results have also highlighted the necessity for early intervention with combinations of lifestyle and/or dietary pharmaceutical medication to modulate the hypothalamic–pituitary–ovary axis.

## Impact of *JCR:LA-cp* Rat Model to Translational Outcomes for Obesity

One measure of impact for the appropriateness of animal models to research is the usefulness of preclinical outcomes for clinical translation. Perhaps the most successful application of the *JCR:LA-cp* rat model has been its suitability to study the progression of metabolic disease from initial stages of overnutrition (without the need for dedicated high caloric diets), resulting in the development of insulin resistance through to the phenotypic complications of hyperinsulinemia and hallmark conditions of the MetS. Many of the advances discussed in this review have made an impact to a better understanding of the human clinical condition. One such example is the revelation of how the intestine integrates and coordinates whole body lipid metabolism more prominently than previously appreciated (114). The discovery that the intestine will contribute to dyslipidemia through unregulated overproduction of lipids has been confirmed clinically in those with insulin resistance and T2D (115, 116). This in turn has provided a new platform for modes of action of different classes of pharmaceutical compounds; including intestinal cholesterol transporter inhibition and incretion blockade. At the same time, the understanding of remnant cholesterol metabolism and how this relates to early conditions of childhood obesity has also come to the fore in the context of potential subclinical risk of CVD (117, 118).

The vision for this research sector will be to continue to strive for ways to aid the younger generation to become more aware of the comorbidities of obesity in childhood, and how they will progress into adulthood. In order to achieve this, we will have to target methodologies that identify metabolic risk of obesity in the younger generation that can have usefulness in the clinic and beyond.

## Author Contributions

Introduction to the clinical problem: SP, WP, and JR. Obesity: testing the “thrifty gene” hypothesis of adaptation to dietary energy intake: AD and WP. Characterization of the altered immune function in obesity using the *JCR:LA-cp* rat model: modulation with age and dietary fat: CF and MR. Inflammation, stroke, and infection in obesity: JP and SA. Development of dyslipidemia during obesity and hyperinsulinemia: overproduction of lipids by the intestine and bioactive trans-fatty acids: RM, MJ-S, and SP. Arteriogenesis is mediated by microRNA in the cardiovasculature and is associated with intestinal lymphatic lipoproteins in the metabolic syndrome: RH and PR. Early intimal atherogenesis, arterial lipid retention, and novel therapeutical targets: RM and SP. Establishing *JCR:LA-cp* rodent rat as a model of spontaneous left ventricular heart dysfunction: FB, SK, and SP. The female *JCR:LA-cp* rat as a model of polycystic ovary syndrome (PCOS) and cardiometabolic risk: DV and AD. Impact of *JCR:LA-cp* rat model to translational outcomes for obesity: SP.

## Conflict of Interest Statement

The authors declare that the research was conducted in the absence of any commercial or financial relationships that could be construed as a potential conflict of interest.
